# The Effects of Industry Sponsorship on Comparator Selection in Trial Registrations for Neuropsychiatric Conditions in Children

**DOI:** 10.1371/journal.pone.0084951

**Published:** 2013-12-23

**Authors:** Adam G. Dunn, Kenneth D. Mandl, Enrico Coiera, Florence T. Bourgeois

**Affiliations:** 1 Centre for Health Informatics, Australian Institute of Health Innovation, University of New South Wales, Sydney, New South Wales, Australia; 2 Children’s Hospital Informatics Program at Harvard-MIT Health Sciences and Technology, Boston Children’s Hospital, Boston, Massachusetts, United States of America; 3 Division of Emergency Medicine, Boston Children’s Hospital, Boston, Massachusetts, United States of America; 4 Department of Pediatrics, Harvard Medical School, Boston, Massachusetts, United States of America; University of British Columbia, Canada

## Abstract

Pediatric populations continue to be understudied in clinical drug trials despite the increasing use of pharmacotherapy in children, particularly with psychotropic drugs. Most pertinent to the clinical selection of drug interventions are trials directly comparing drugs against other drugs. The aim was to measure the prevalence of active drug comparators in neuropsychiatric drug trials in children and identify the effects of funding source on comparator selection. We analyzed the selection of drugs and drug comparisons in clinical trials registered between January 2006 and May 2012. Completed and ongoing interventional trials examining treatments for six neuropsychiatric conditions in children were included. Networks of drug comparisons for each condition were constructed using information about the trial study arms. Of 421 eligible trial registrations, 228 (63,699 participants) were drug trials addressing ADHD (106 trials), autism spectrum disorders (47), unipolar depression (16), seizure disorders (38), migraines and other headaches (15), or schizophrenia (11). Active drug comparators were used in only 11.0% of drug trials while 44.7% used a placebo control and 44.3% no drug or placebo comparator. Even among conditions with well-established pharmacotherapeutic options, almost all drug interventions were compared to a placebo. Active comparisons were more common among trials without industry funding (17% vs. 8%, p=0.04). Trials with industry funding differed from non-industry trials in terms of the drugs studied and the comparators selected. For 73% (61/84) of drugs and 90% (19/21) of unique comparisons, trials were funded exclusively by either industry or non-industry. We found that industry and non-industry differed when choosing comparators and active drug comparators were rare for both groups. This gap in pediatric research activity limits the evidence available to clinicians treating children and suggests a need to reassess the design and funding of pediatric trials in order to optimize the information derived from pediatric participation in clinical trials.

## Introduction

Pediatric populations continue to be understudied in clinical drug trials despite national and global initiatives to increase the pediatric evidence guiding clinical practice[1-4]. In contrast, there is increasing use of pharmacotherapy in children, particularly with psychotropic drugs[5-8]. Antipsychotic and antidepressant use in the United States, for example, more than doubled from 8.6 to 39.4 per 1000 children and from 9.4 to 21.3 per 1000 children, respectively, over a ten year period beginning in the mid-nineties[9,10]. However, the number of trials supporting use of these and other psychotropic medications in children is small, resulting in widespread off-label prescribing among children with neuropsychiatric conditions that is often supported by no or inconclusive evidence at best[9,11,12]. 

Most pertinent to doctors’ clinical selections of drug interventions are trials directly assessing drugs against other drugs – active comparisons of an interventional drug to an active drug comparator[13-15]. These comparative effectiveness studies are particularly important when multiple drugs are already well established in clinical practice. For example, epilepsy is treated primarily with pharmacotherapy and has seen a recent rapid rise in medication options with ten anti-epileptic medications receiving market approval in the United States since 2000. In contrast, conditions like autism spectrum disorders, which have little evidence for medication effectiveness, may require placebo controlled trials to identify the first efficacious drugs[16]. Placebo-controlled trials also play an important role in studying conditions with known placebo effects or with uncertainty around the efficacy of existing drugs[17,18]. 

Nonetheless, analyses have shown that placebo-controlled trials and trials without comparators are more likely to yield results favoring the agent under investigation, potentially making treatments appear more effective than they really are[19-21]. Funding source may also play a role, with industry funded trials more likely to avoid active comparators[21-23]. 

The aims of the study were to measure the prevalence of active drug comparators in neuropsychiatric drug trials in children and identify the effects of funding source on comparator selection. We hypothesized that active comparators would be used less frequently in industry funded trials than in non-industry funded trials.

## Methods

### Trial selection

We identified eligible clinical trials using ClinicalTrials.gov, a publicly available, web-based trial registry[24]. For each study, standard information is provided that includes the condition and interventions studied, the study phase and endpoints, characteristics of the subjects enrolled, anticipated number of subjects, funding sources supporting the trial, and current trial status. We selected all trials that were pediatric, studied one or more interventions for the treatment of a neuropsychiatric condition, and started on or after January 1, 2006. All data were downloaded from the registry on May 2, 2012. Pediatric trials were defined as trials in which the mid-point of the age range of enrolled subjects was less than 18 years[1]. We defined drug trials as those studying an agent listed in the WHO Anatomical Therapeutic Chemical classification system[25]. 

Trials were categorized as entirely industry-funded, partially industry-funded, or receiving no industry funding based on sponsor information provided in the registry. Any trial labeled as having an industry sponsor and no other was defined as an entirely industry-funded trial, and partially industry-funded trials were those labeled with an industry sponsor and at least one other type of sponsor. All other combinations were defined as non-industry trials. Trials that were withdrawn or terminated prior to completion were excluded. 

**Figure 1:**
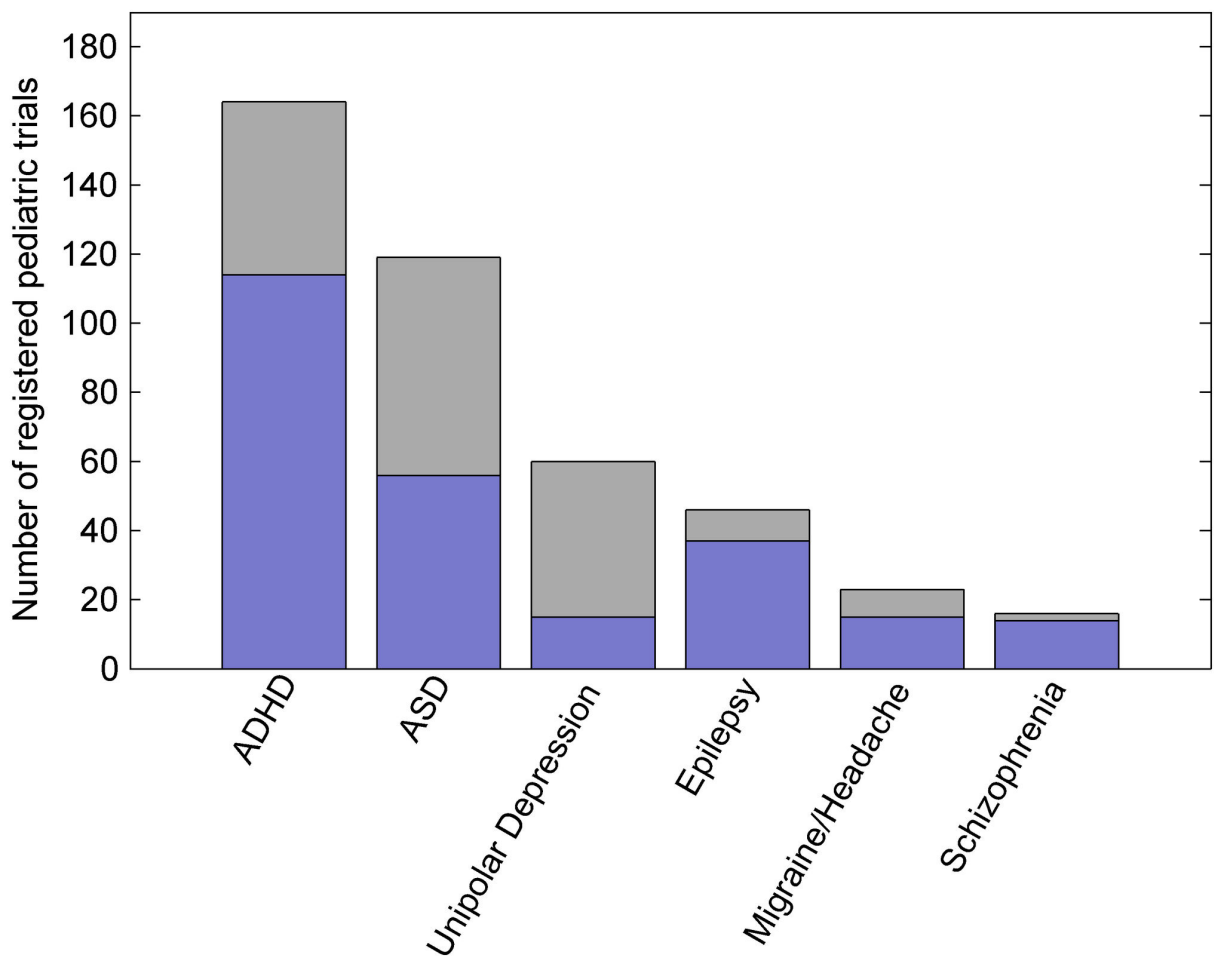
Neuropsychiatric interventional studies involving children, registered on clinicaltrials.gov January 2006 to May 2012. The set of pediatric interventional studies registered on ClinicalTrials.gov that met the inclusion criteria and included at least one drug intervention (blue); or only non-drug interventions (grey). Abbreviations: ADHD: attention deficit hyperactivity disorder; ASD: autism spectrum disorder.

Among the remaining trials, those studying the six neuropsychiatric conditions with the largest number of drug trials were included: attention deficit hyperactivity disorder (ADHD), autism spectrum disorders, unipolar depression, seizure disorders, migraines and other headaches, and schizophrenia (Figure 1). The five other conditions identified in the set had too few registered drug trials to enable meaningful analysis and these trials were excluded. 

### Network Construction

For each trial, we extracted information on the interventions and study arms and identified the experimental and comparator treatments. All eligible trials contributed to the structure of condition-specific networks in which nodes represent interventions and connections represent direct comparisons between interventions[16,22,26,27]. Comparators were classified as active if the trial registration listed another specific therapeutic intervention, as a placebo if a placebo (or synonym) was described, or a control otherwise. Crossover studies with a placebo control arm were categorized as placebo-controlled trials. Trials involving different doses of a single drug without another active or placebo comparator were included in the networks as single arm trials. Multiple-arm trials with a placebo and at least one active comparator were classified as active comparator trials. 

Since trials were used as the denominator in the networks, three trials with more complicated designs could not be completely coded. In these cases, the first comparison listed in the trial record was included in the network. 

### Analysis

Analysis of the networks was based on three metrics that quantify biases in the distribution of interventions and their comparisons. Each of the network metrics is considered relative to a standard baseline, which normalizes for differences in size and density, and suggests levels of significance for the metrics[28]. The size and density of a network represents how many interventions were considered for the conditions, and how frequently those interventions are tested against the other interventions.

*Degree centralization* indicates whether some treatments are chosen as comparators more often than others[29]. Degree centralization increases from zero to one as the network approaches a star-shaped pattern, where one intervention is the preferred comparator in every study. The degree centralization percentile is calculated using a baseline of network permutations with the same size and density as the observed network. The simulated networks represent networks in which there is no underlying bias in comparator selection, and thus reflects the extremity of the observed centralization score[28,30].

The probability of interspecific encounter (PIE) measures the diversity of a treatment and is calculated as the probability that any two randomly selected treatment groups in the network are treated with two different interventions[16,31,32]. PIE scores increase as trials are distributed more evenly across a large number of treatments. Conversely, trials with smaller numbers of treatments and in which certain interventions are studied more often than others will have a lower PIE score. Scores between 0.7 and 0.8 represent poor diversity, and scores lower than 0.7 represent very low diversity with a strong preference for specific treatments. The PIE’ is the raw PIE score normalized by the maximum possible PIE score for the number of trials in the network.

The role of funding in the networks was analyzed in terms of *funding exclusivity*, which measures the separation of funding across the set of drugs examined in each condition. For each drug, if every trial is conducted with only industry funding, or only non-industry funding, then that drug is considered to be funded exclusively by one funding type. The funding exclusivity is then defined as the proportion of all drugs across a condition funded exclusively by one funding type (either industry or non-industry but not both). As with the other measures, funding exclusivity is considered relative to a simulated baseline of permutations, which indicates the extremity of the score beyond what random chance is expected to produce.

## Results

**Figure 2:**
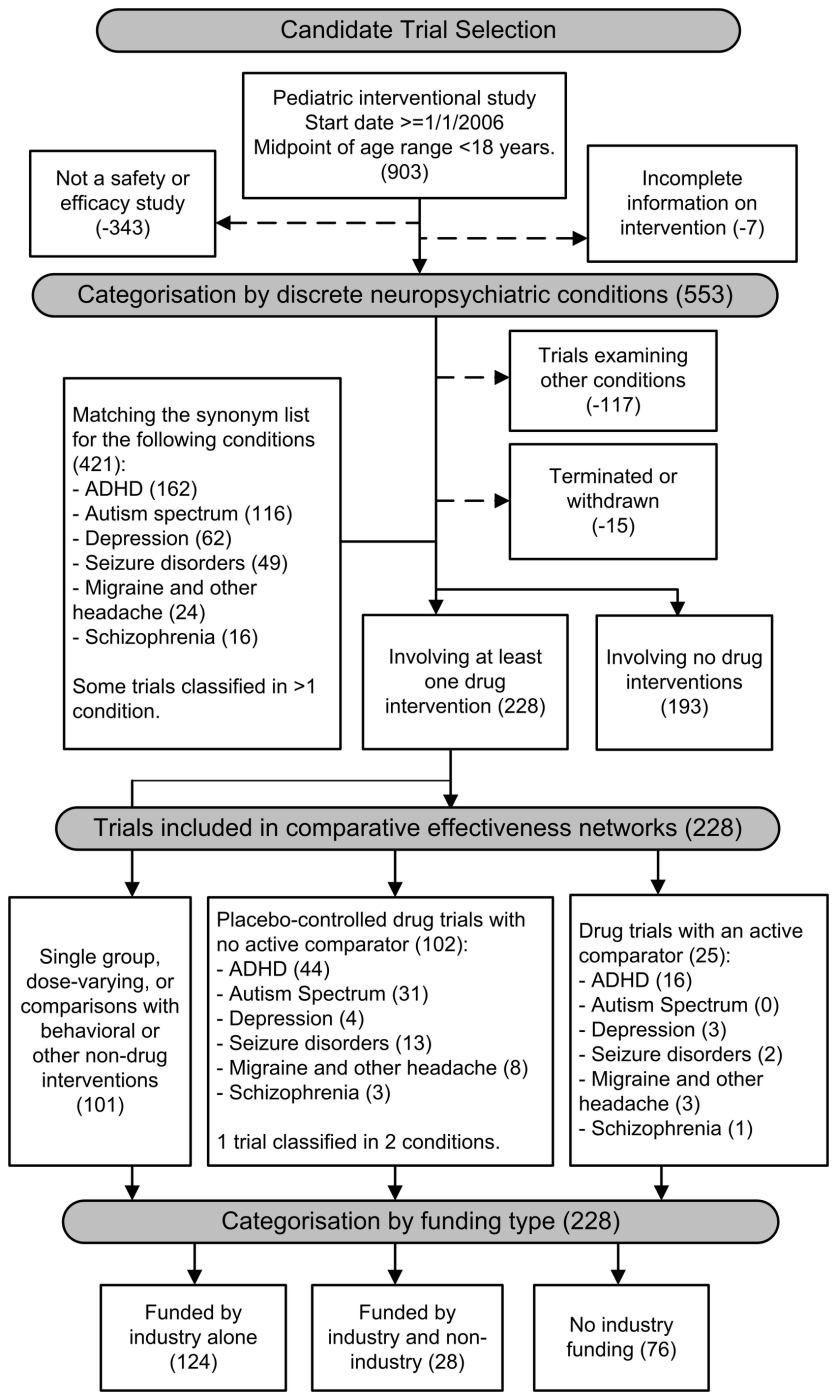
Selection and classification of clinical drug trials. The trial selection and classification used in the analyses are also classified by funding type.

Drugs were studied in 228 trials, corresponding to 54.2% of 421 trials meeting the inclusion criteria and conditions (see Table S2 for trials and Figures S1 to S6 for details of trials examining interventions other than drugs). Of the 228, 11.0% (25/228) employed an active drug comparator, representing the overall number of active comparator trials. Another 44.7% (102/228) used a placebo control and 44.3% (101/228) were single-group assignments or trials comparing drugs with behavioral or other non-drug interventions (Figure 2). 

### Drug Trial Characteristics

Study characteristics are presented in Table 1. The total number of anticipated or actual enrolments across the 228 trials was 63,699. The median enrolment in trials was low, ranging from 59.5 for seizure disorders, to 201 for schizophrenia. Fewer than 10 different drug interventions were studied for unipolar depression and schizophrenia. For ADHD, unipolar depression, migraines and other headaches, and schizophrenia, 20% or more of trials focused on a single drug. 

**Table 1:** Characteristics of pediatric drug trials registered in ClinicalTrials.gov for selected conditions.

	Attention Deficit Hyperactivity Disorder	Autism Spectrum Disorders	Seizure Disorders	Unipolar Depression	Migraines and Headaches	Schizophrenia
Drug trials, No.	106	47	38	16	15	11
Total enrolment (median)**^*a*^**	18385 (135)	3690 (60)	4231 (59.5)	3251 (160)	7390 (150)	2385 (201)
Phase III/IV trials, No. (%)**^*b*^**	87/101 (86%)	24/43 (56%)	24/36 (67%)	10/15 (67%)	10/12 (83%)	11/11 (100%)
Trials with safety outcomes, No. (%)**^*c*^**	60 (57%)	29 (62%)	20 (53%)	7 (44%)	6 (40%)	6 (54%)
Unique drug interventions, No.	22	25	22	9	12	6
Most common drug tested	methylphenidate	aripiprazole	levetiracetam	desvenlafaxine	rizatriptan	aripiprazole
-No. (%)	41 (39%)	8 (17%)	7 (18%)	6 (38%)	3 (20%)	4 (36%)
**Funding source**						
-Entirely industry-funded, No. (%)	57 (54%)	12 (26%)	26 (68%)	14 (88%)	7 (47%)	9 (82%)
-Partially industry-funded, No. (%)	13 (12%)	10 (21%)	2 (5.3%)	1 (6.2%)	1 (6.7%)	1 (9.1%)
-No industry funding, No. (%)	36 (34%)	25 (53%)	10 (26%)	1 (6.2%)	7 (47%)	1 (9.1%)

^a^ Median enrolment is from expected enrolment for trials in the recruiting phase and actual enrolment for completed trials.

^b^ Not all trials specified information about the clinical trial phase; the denominator is the number of trials with this information registered.

^c^ Trials for which the registration included one or more measurable outcomes labelled as a safety outcome.

**Figure 3:**
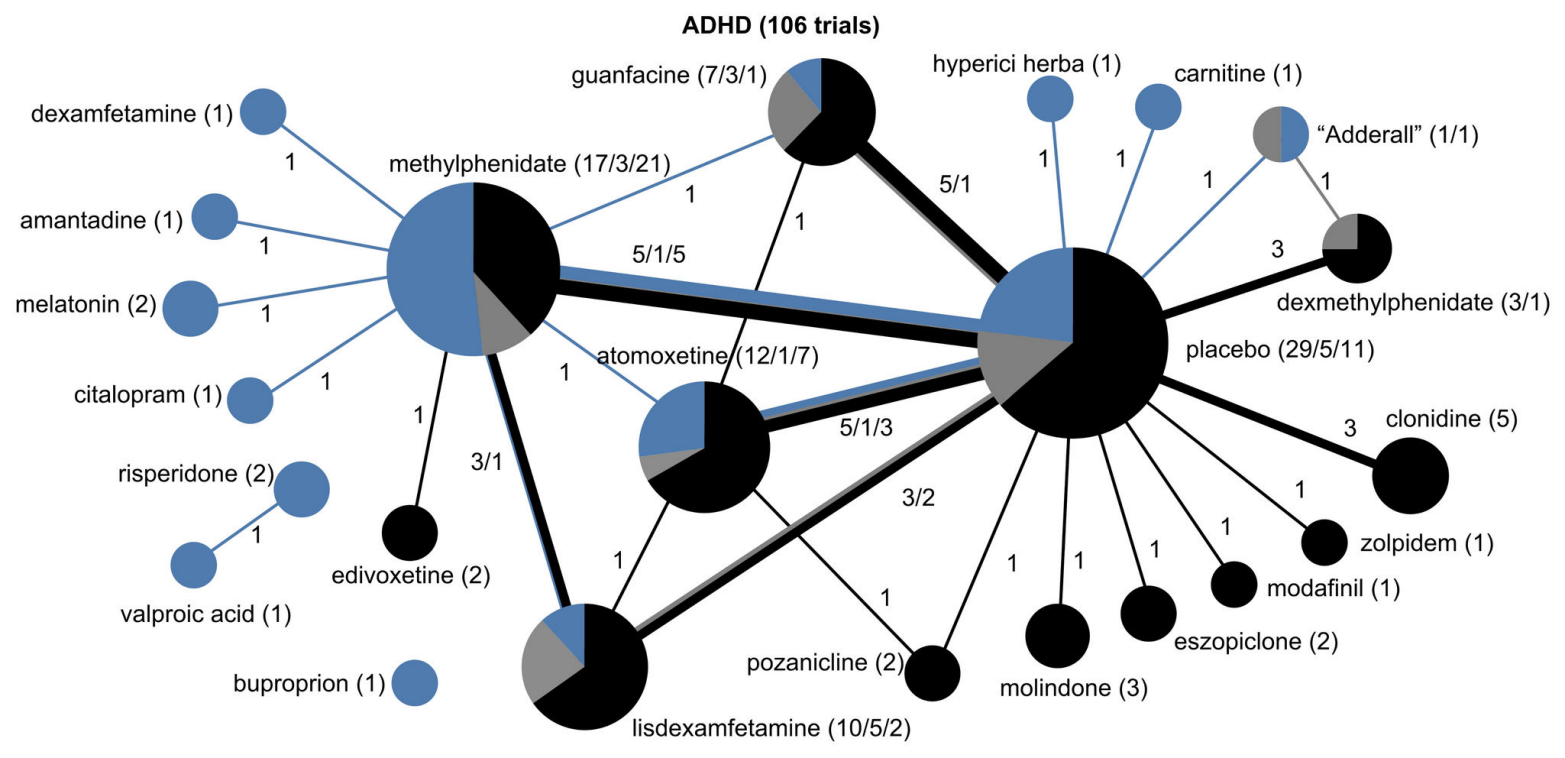
The drug treatment network for ADHD, indicating funding source. The network of drug treatments for 106 ADHD trials, where the areas of circles are proportional to the total number of trials examining the treatments, and the thickness of the lines are proportional to the number of active comparisons between drugs or with placebo. Black represents industry-funded trials; grey represents trials partially funded by industry; and blue represents non-industry funding. The numerical labels are the number of trials in each group, matching the colors.

**Figure 4:**
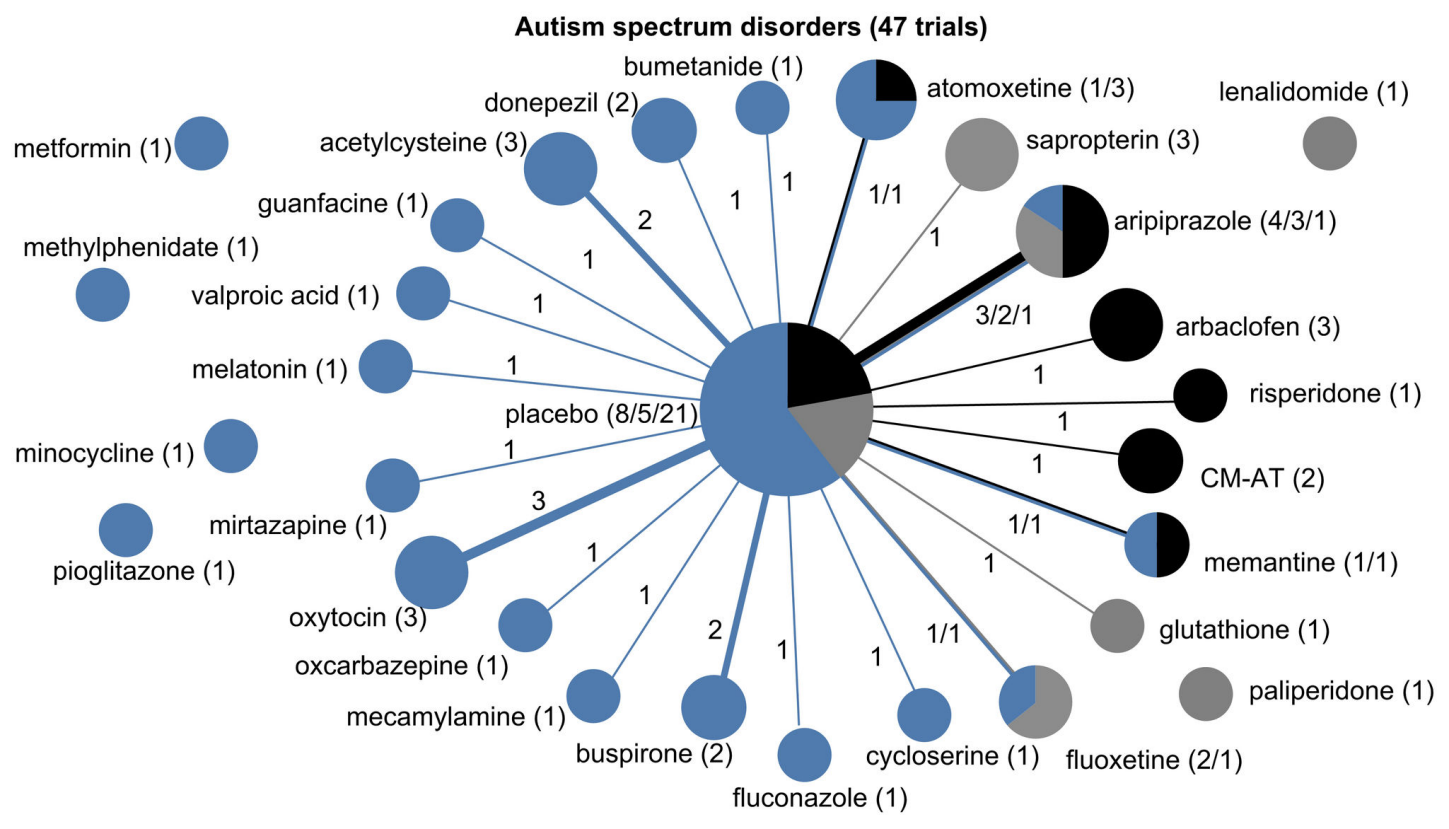
The drug treatment network for autism spectrum disorders, indicating funding source. The network of drug treatments for 47 autism spectrum disorder trials, where the areas of circles are proportional to the total number of trials examining the treatments, and the thickness of the lines are proportional to the number of active comparisons between drugs or with placebo. Black represents industry-funded trials; grey represents trials partially funded by industry; and blue represents non-industry funding. The numerical labels are the number of trials in each group, matching the colors.

**Figure 5:**
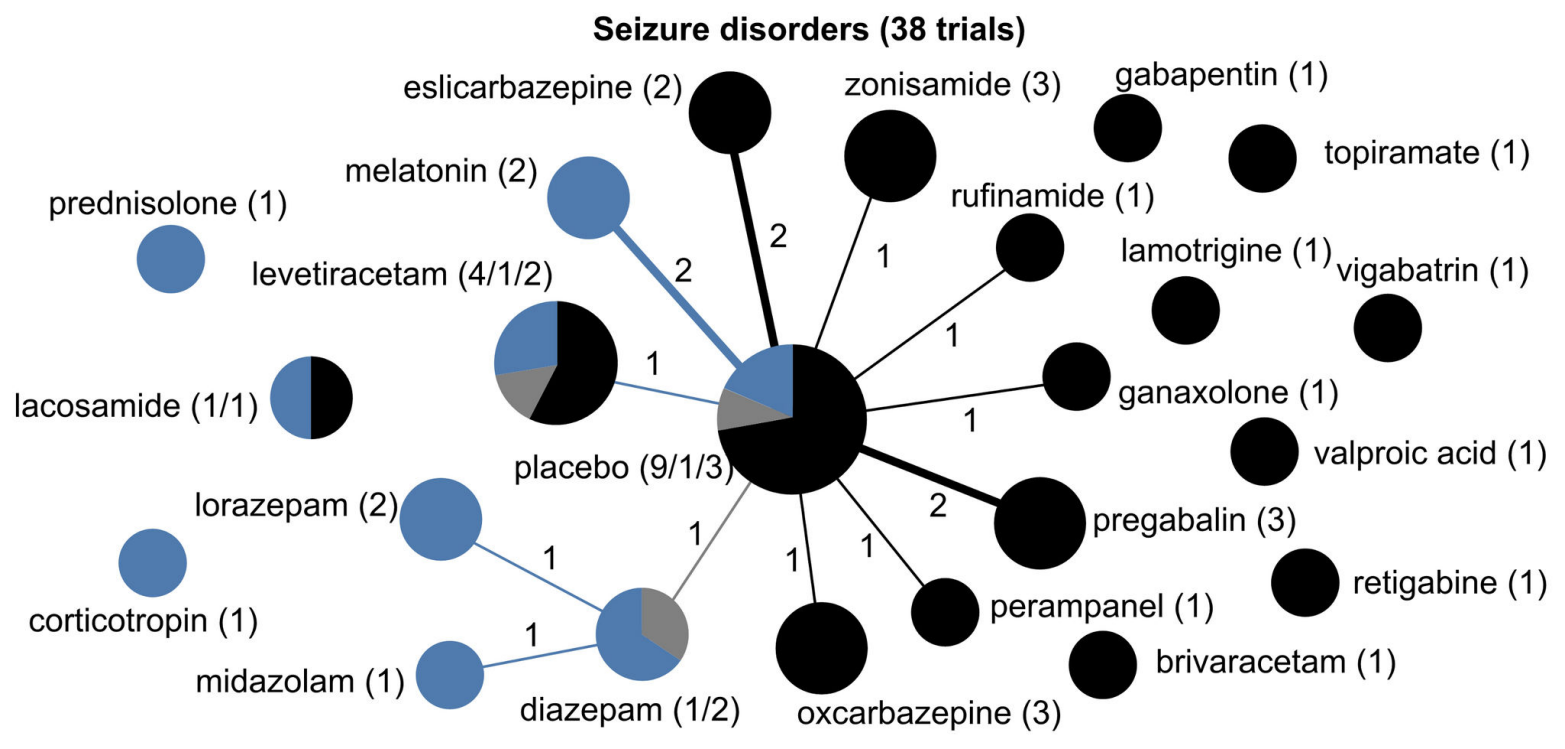
The drug treatment network for seizure disorders, indicating funding source. The network of drug treatments for 38 seizure disorder trials, where the areas of circles are proportional to the total number of trials examining the treatments, and the thickness of the lines are proportional to the number of active comparisons between drugs or with placebo. Black represents industry-funded trials; grey represents trials partially funded by industry; and blue represents non-industry funding. The numerical labels are the number of trials in each group, matching the colors.

**Figure 6:**
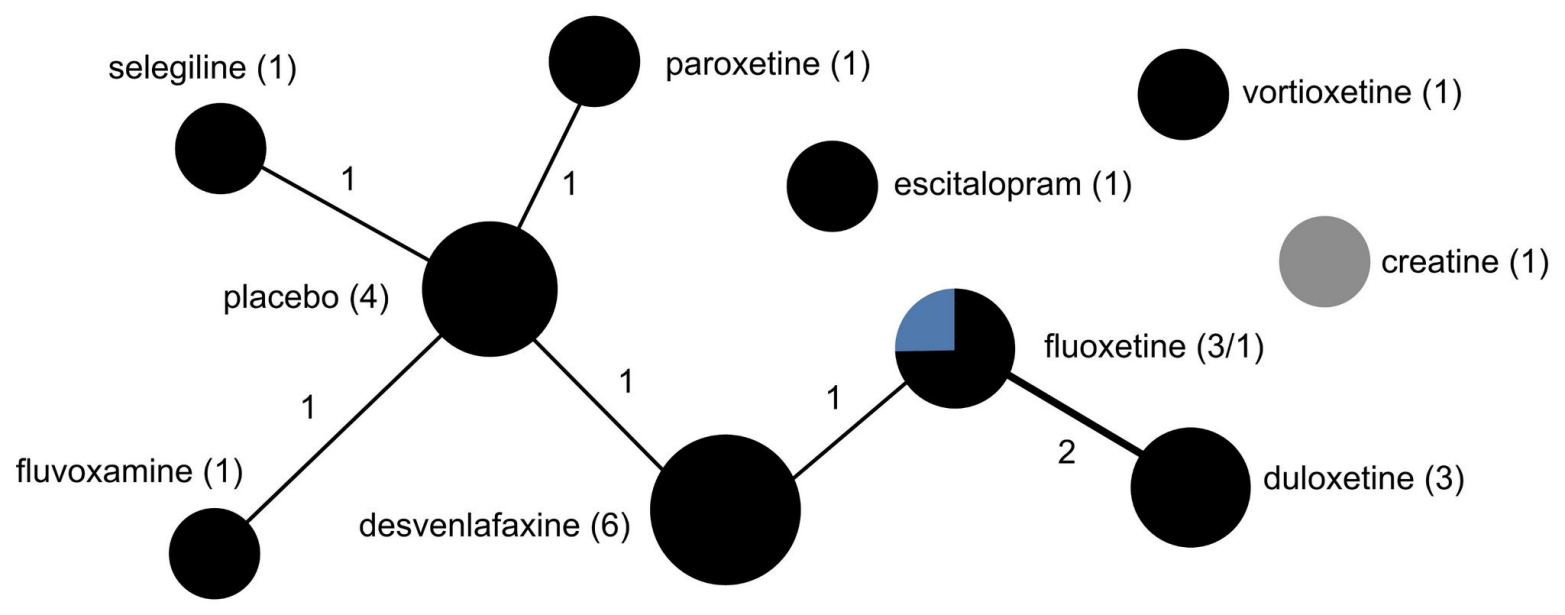
The drug treatment network for unipolar depression, indicating funding source. The network of drug treatments for 16 unipolar depression trials, where the areas of circles are proportional to the total number of trials examining the treatments, and the thickness of the lines are proportional to the number of active comparisons between drugs or with placebo. Black represents industry-funded trials; grey represents trials partially funded by industry; and blue represents non-industry funding. The numerical labels are the number of trials in each group, matching the colors.

**Figure 7:**
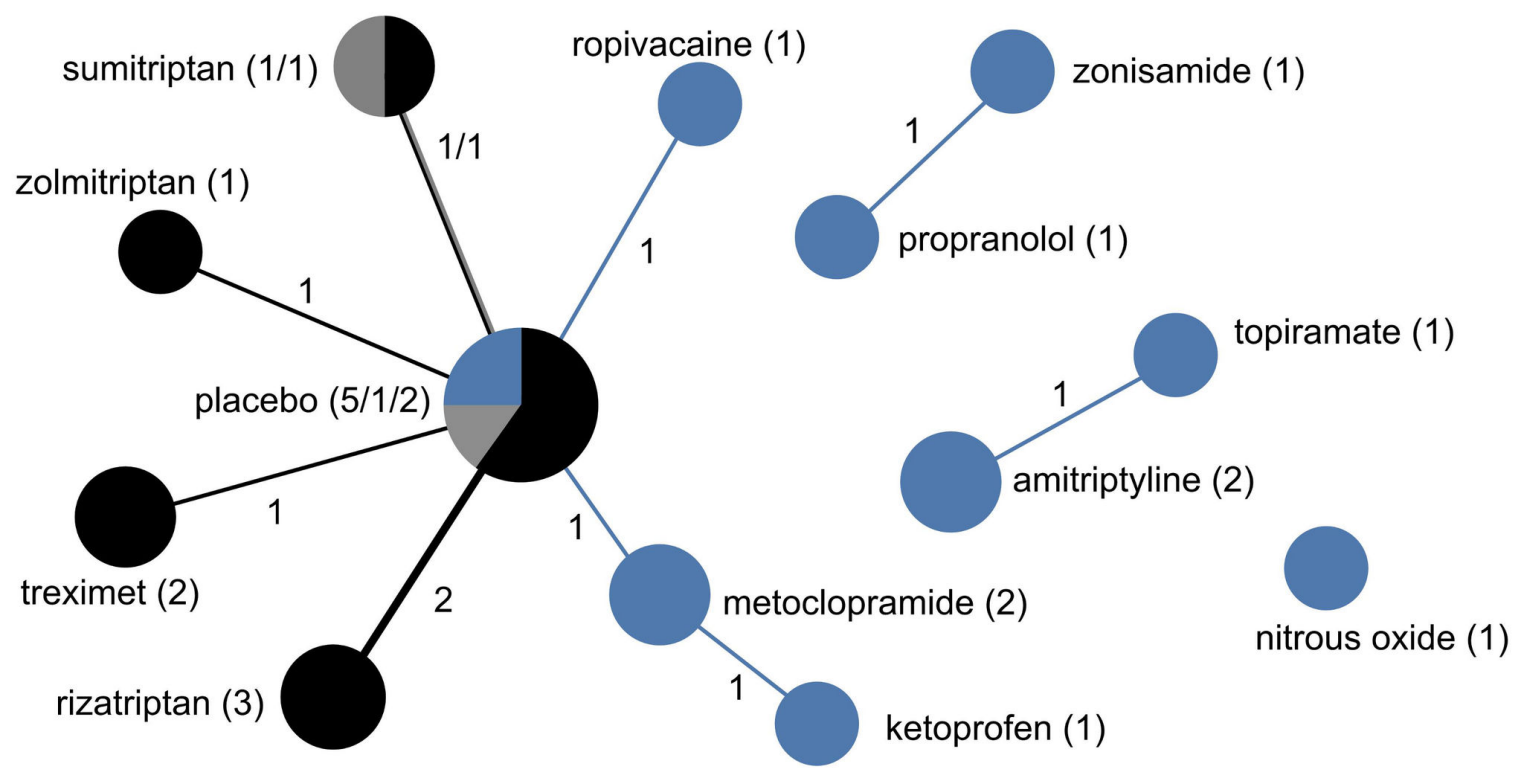
The drug treatment network for migraines and other headaches, indicating funding source. The network of drug treatments for 15 migraines and other headaches trials, where the areas of circles are proportional to the total number of trials examining the treatments, and the thickness of the lines are proportional to the number of active comparisons between drugs or with placebo. Black represents industry-funded trials; grey represents trials partially funded by industry; and blue represents non-industry funding. The numerical labels are the number of trials in each group, matching the colors.

**Figure 8:**
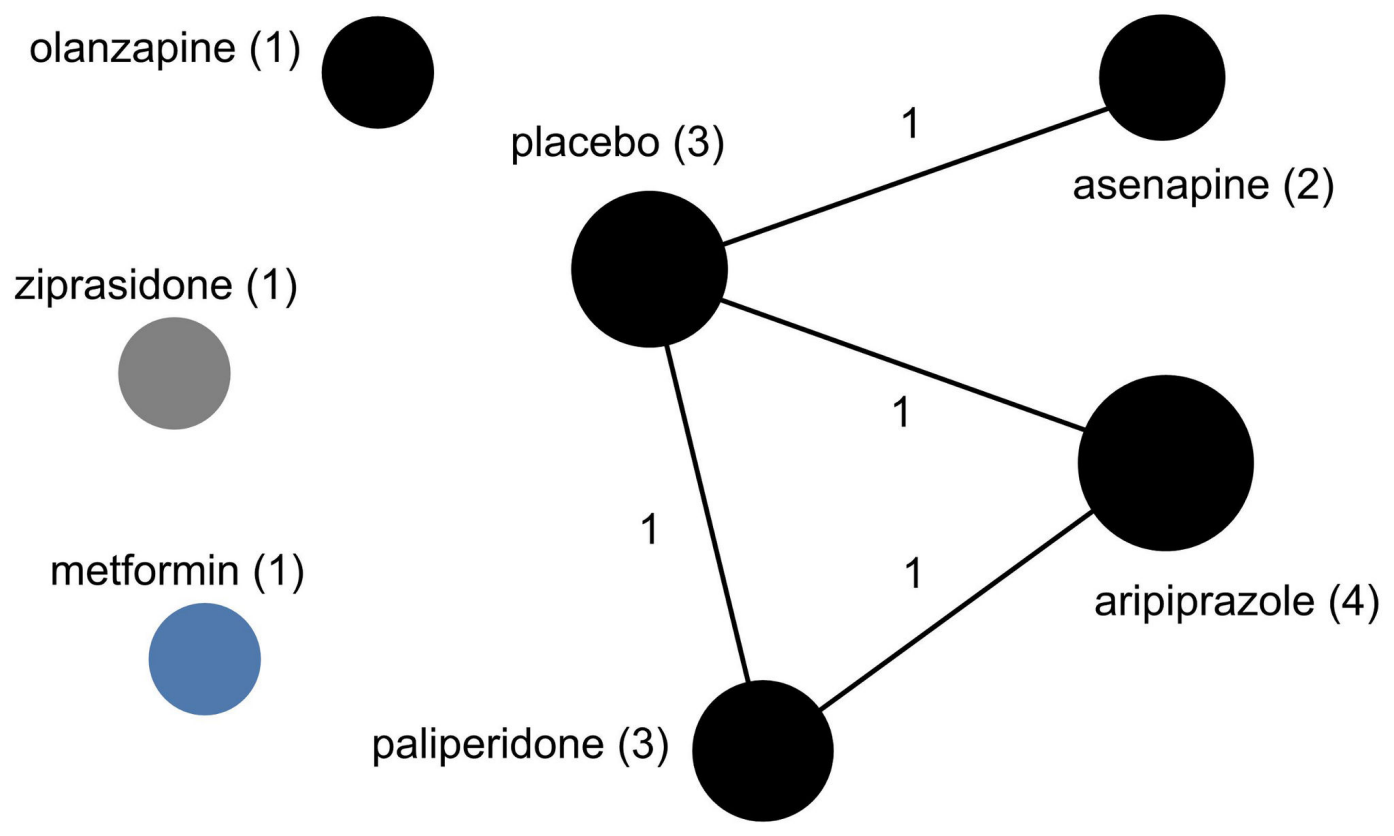
The drug treatment network for schizophrenia, indicating funding source. The network of drug treatments for 11 schizophrenia trials, where the areas of circles are proportional to the total number of trials examining the treatments, and the thickness of the lines are proportional to the number of active comparisons between drugs or with placebo. Black represents industry-funded trials; grey represents trials partially funded by industry; and blue represents non-industry funding. The numerical labels are the number of trials in each group, matching the colors.

Drug treatment networks are illustrated for the six conditions (Figures 3 to 8). Among the drug trials, the predominant source of funding was industry, with as many as 88% of unipolar depression trials and 82% of schizophrenia trials funded exclusively by industry. Autism spectrum disorders represented the only condition in which the majority of trials (53%) were funded without industry sponsors. 

### Comparators and Treatment Networks for Drug Trials

Less than 10% of drug trials employed an active comparator among trials examining autism spectrum disorders, seizure disorders, and schizophrenia (Table 2). The majority of drug trials for autism spectrum disorders and migraines and other headaches employed a placebo comparator, while most trials for unipolar depression, seizure disorders, and schizophrenia were single group studies. 

**Table 2:** Network metrics associated with the drug-based comparative effectiveness networks.

	ADHD	Autism Spectrum Disorders	Seizure Disorders	Unipolar Depression	Migraines and Headaches	Schizophrenia
Number of included drug trials	106	47	38	16	15	11
Number of drug trials involving an active drug comparator (%)	16 (15%)	0 (0%)	2 (5.3%)	3 (19%)	3 (20%)	1 (9.1%)
Network size (drugs with active and placebo comparisons)	23	26	23	10	13	7
Density of the network	0.15	0.08	0.07	0.13	0.15	0.18
Raw degree centralization	0.56	0.71	0.43	0.36	0.45	0.38
Degree centralization percentile**^*a*^**	≈100%	=100%	≈100%	87%	99%	60%
Probability of interspecific encounter (PIE)	0.81	0.74	0.81	0.88	0.87	0.82
Normalised PIE (PIE’)	0.84	0.76	0.81	0.90	0.90	0.82

^a^ The percentile is equal to 100% when the degree centralization is known to be uniquely high, and approximately 100% when after 100,000 simulated networks, no higher value was encountered amongst the random baseline set of networks.

All six conditions featured strong indications of lack of diversity and high centralization, reflecting a tendency for trials to focus on select drugs and to preferentially choose certain comparators while avoiding others (Table 2). This was most pronounced for autism spectrum disorder trials, which formed a perfectly star-shaped network with every drug compared to a placebo or without a control (Figure 4). Seizure disorder trials were similar (Figure 5), with all but two of the trials comparing a drug intervention to a placebo or no control. While ADHD trials had a relatively higher proportion of active comparator trials, the treatment network also demonstrated low diversity and high centralization because of a tendency for trials to focus on a limited number of drugs and to preferentially select one of a few drug comparators (Figure 3). 

### Role of Funding on Drug and Comparator Selection

Industry and non-industry funded trials differed in terms of the drugs studied and the comparators employed in trials (Table 3). Drugs were generally studied exclusively by industry or non-industry sources, with 73% (61/84) of drugs studied only by either industry or non-industry sponsors (see Table S1). Unipolar depression included the highest proportion of drugs examined only by industry (78%; 7/9) and migraines and other headaches the highest proportion examined only by non-industry (67%; 8/12). High levels of funding exclusivity were evident relative to a random baseline in all conditions except unipolar depression (which was dominated by industry-funded trials allowing for only two unique funding exclusivity scores). Amongst the other five conditions, four were found to have funding exclusivity percentiles above 97% when compared to the permutation baseline, indicating a strong presence of bias. 

**Table 3:** Funding characteristics of drug-based clinical trials.

	ADHD (n=106)	Autism Spectrum Disorders (n=47)	Seizure Disorders (n=38)	Unipolar Depression (n=16)	Migraines and Headaches (n=15)	Schizophrenia (n=11)
Trials employing an active drug comparator, No. (%)	16 (15%)	0	2 (5.3%)	3 (19%)	3 (20%)	1 (9.1%)
- Entirely industry-funded	7 (6.6%)	0	0	3 (19%)	0	1 (9.1%)
- Partially industry-funding	1 (0.9%)	0	0	0	0	0
- No industry funding	8 (7.5%)	0	2 (5.3%)	0	3 (20%)	0
Trials employing a placebo comparator only, No. (%)	44 (42%)	31 (66%)	13 (34%)	4 (25%)	8 (53%)	3 (27%)
- Entirely industry-funded	29 (27%)	8 (17%)	9 (24%)	4 (25%)	5 (33%)	3 (27%)
- Partially industry-funding	4 (3.8%)	5 (11%)	1 (2.6%)	0	1 (6.7%)	0
- No industry funding	11 (10%)	18 (38%)	3 (7.9%)	0	2 (13%)	0
Single group and other drug trials with no placebo or active drug comparator, No. (%)**^*a*^**	46 (43%)	16 (34%)	23 (61%)	9 (56%)	4 (27%)	7 (64%)
- Entirely industry-funded	21 (20%)	4 (8.5%)	17 (45%)	7 (44%)	2 (13%)	5 (45%)
- Partially industry-funding	8 (7.6%)	5 (11%)	1 (2.6%)	1 (6.2%)	0	1 (9.1%)
- No industry funding	17 (16%)	7 (15%)	5 (13%)	1 (6.2%)	2 (13%)	1 (9.1%)
Funding exclusivity for drugs, (%)**^*b*^**	73% (16/22)	71% (17/25)	68% (7/9)	78% (7/9)	92% (11/12)	83% (5/6)
Funding exclusivity for active comparisons, (%)**^*b*^**	85% (11/13)	NA (0/0)	100% (2/2)	100% (2/2)	100% (3/3)	100% (1/1)

^a^ Includes trials comparing drugs with behavioral or other non-drug interventions.

^b^ Funding exclusivity represents the percentage of drugs and active comparisons whose constituent trials are funded exclusively by industry or by non-industry sources.

Fewer than half (12/25) of the active comparator trials received industry funding. Overall, trials with industry funding were less likely to use active drug comparators compared to trials funded by non-industry sources (8.0% [12/152] vs. 17.1% [13/76], p-value for chi-square = 0.04). Among active comparator trials, 90% (19/21) of the unique active comparisons were funded exclusively by industry or non-industry funders. The treatment network for ADHD demonstrated the most even distribution of funding among active comparator trials, although industry remained less likely to use active comparators compared to non-industry sources (11% of industry-funded trials vs. 22% of non-industry-funded trials). 

## Discussion

Active comparator trials in pediatric drug research remain limited across a number of neuropsychiatric conditions and drug trials funded by industry are less likely to involve active drug comparisons than their non-industry counterparts. This is most pronounced in conditions that have few known pharmaceutical treatments, but is also the standard for diseases treated primarily with medications in which clinicians already have a number of well-established drug options. We also found substantial differences between the drugs and comparators chosen by industry and non-industry groups, showing strong preferences for studying specific drugs and drug comparisons.

Prior work has demonstrated concerning findings with respect to the medical evidence guiding the clinical care of children. First, there appears to be a paucity of clinical research—and particularly randomized controlled trials—conducted in pediatric relative to adult patients[1,33-35]. This pattern exists across a number of disease conditions and has been attributed chiefly to the smaller disease burden among children as well as issues around the cost and complexity of conducting research in children, all of which translate into a lower potential for profitability[36-38]. Second, the research that is conducted in children appears to be of only moderate quality with pediatric studies demonstrating high risks of bias in study design and outcome reporting and insufficient safety assessments[12,30,39,40]. The current study adds to this body of findings by demonstrating a gap in the pediatric research portfolio, specifically with respect to selection of study drugs and drug comparisons.

There are a number of scenarios that necessitate placebo-controlled trials, such as conditions with limited pharmaceutical options, or where there is uncertainty regarding the true efficacy of standard options compared to placebo[41-43]. However, these arguments do not fully explain our findings that pediatric drug trials are conducted predominantly with placebos or no controls in the study of ADHD, seizure disorders, unipolar depression, schizophrenia, and migraines and other headaches. In each of these conditions, less than a quarter of the trials were active comparator trials. Although it is difficult to estimate what the appropriate rate of active-comparator trials should be, examining the number of condition-specific drugs that have Federal Drug Agency (FDA) approval in the United States provides a gauge of the existing availability and establishment of drug options. For example, among the antiepileptic drugs we examined, 57% are FDA approved for seizure management, indicating that for the majority of drugs, their superiority to placebo have been previously established with reasonable certainty. Similarly, for ADHD, unipolar depression, migraines and other headaches, and schizophrenia, the rates of FDA approval for the studied drugs indicate that there are a number of well-established existing drug options (30%, 60%, 42%, and 83%, respectively). Even given the potential benefit of including placebo trials within the research portfolio for a condition, one would expect a greater proportion of active-comparator trials based on the amount of existing evidence supporting the efficacy of available drugs.

We found that industry-funded trials were less likely to choose active comparators in their trials compared to non-industry studies, and that they focused on drugs and drug comparisons that were different than their non-industry counterparts. This type of bias in the selection and avoidance of specific drugs and drug comparisons by manufacturers has been previously described for antirheumatic drugs, antipsychotics, and anti-fungal agents[22,44,45]. Drug companies focus primarily on the drugs that they manufacture, which tend to be newer and more expensive agents[44,46]. When comparators are employed, they may choose inactive or placebo controls as they are most likely to yield favorable results that can be readily used for marketing purposes[47]. Alternatively, specific comparators may be favored because they are considered suboptimal, again ensuring favorable results for the drug under study. It is likely that regulatory agencies have contributed to the preponderance of placebo-controlled trials since active-comparator trials are not required as part of the drug approval process[14,47]. However, it has also been suggested that the choice of comparators by industry sponsors represents calculated marketing decisions to augment drug effectiveness estimates and promote the agents under study[22,44,46]. Unfortunately, this type of biased comparator selection limits the breadth of evidence available on the true value of a drug in the context of all available options.

Our study is limited to the data available in the registry and we were unable to verify the completeness or accuracy of the data provided. However, ClinicalTrials.gov is the largest and most widely used trial registry and performs internal data quality and verification procedures prior to posting the information submitted by investigators. It is also unlikely that unregistered trials systematically included a higher rate of active comparisons or studied drugs that were underrepresented in the current research networks. 

## Conclusions

In this study across six neuropsychiatric conditions in children, we found a dearth of trials examining the comparative effectiveness of drugs, and high proportions of trials designed with either no control or a placebo. This was apparent across the funding spectrum, but more pronounced among trials with industry funding. The lack of active comparisons spanned most neuropsychiatric conditions and was evident both for conditions in which drug therapy remains exploratory (e.g. autism), and for those in which drugs are well-established as the primary treatment and are in widespread clinical use (e.g. seizure disorders). The interventions and comparators examined by industry and non-industry funding sources were mostly distinct, suggesting that the evidence base for many approved treatments is derived exclusively by specific types of sponsors. The overall bias against comparative effectiveness trials and the strong funding exclusivity between industry and non-industry funded drugs and drug comparisons may limit the reliability of emerging evidence for the treatment of children with neuropsychiatric conditions, despite the growing use of pharmacotherapy in this area. The mechanisms underlying the design and funding of pediatric trials should be reassessed in order to optimize the information derived from pediatric participation in clinical trials.

## Supporting Information

Table S1 **Drugs examined in neuropsychiatric trials involving children, across six conditions, ordered by prevalence in the set of 228 trials.**
(DOC)

Table S2 **The set of included trials for each of the six conditions.**
(DOC)

Figure S1 **The treatment network for ADHD including interventions other than drugs.** There were 162 trials that met the inclusion criteria for ADHD, illustrated here as a treatment network (inclusive of non-drug trials). Black circles represent drug trials, grey nodes represent non-drug trials. The area of the circles is proportional to the number of trials. Connections represent comparisons in trials. Connection width is proportional to the number of trials in which the comparison was present. The numbers for both circles and connections indicate trial counts.
(TIF)

Figure S2 **The treatment network for autism spectrum disorders including interventions other than drugs** There were 116 trials that met the inclusion criteria for autism spectrum disorders, illustrated here as a treatment network (inclusive of non-drug trials). Black circles represent drug trials, grey nodes represent non-drug trials. The area of the circles is proportional to the number of trials. Connections represent comparisons in trials. Connection width is proportional to the number of trials in which the comparison was present. The numbers for both circles and connections indicate trial counts.
(TIF)

Figure S3 **The treatment for seizure disorders including interventions other than drugs** There were 49 trials that met the inclusion criteria for seizure disorders, illustrated here as a treatment network (inclusive of non-drug trials). Black circles represent drug trials, grey nodes represent non-drug trials. The area of the circles is proportional to the number of trials. Connections represent comparisons in trials. Connection width is proportional to the number of trials in which the comparison was present. The numbers for both circles and connections indicate trial counts.
(TIF)

Figure S4 **The treatment network for unipolar depression including interventions other than drugs.** There were 66 trials that met the inclusion criteria for unipolar depression, illustrated here as a treatment network (inclusive of non-drug trials). Black circles represent drug trials, grey nodes represent non-drug trials. The area of the circles is proportional to the number of trials. Connections represent comparisons in trials. Connection width is proportional to the number of trials in which the comparison was present. The numbers for both circles and connections indicate trial counts.
(TIF)

Figure S5 **The treatment network for migraines and other headaches including interventions other than drugs.** There were 24 trials that met the inclusion criteria for migraines and other headaches, illustrated here as a treatment network (inclusive of non-drug trials). Black circles represent drug trials, grey nodes represent non-drug trials. The area of the circles is proportional to the number of trials. Connections represent comparisons in trials. Connection width is proportional to the number of trials in which the comparison was present. The numbers for both circles and connections indicate trial counts.
(TIF)

Figure S6 **The treatment network for schizophrenia including interventions other than drugs.** There were 24 trials that met the inclusion criteria for schizophrenia, illustrated here as a treatment network (inclusive of non-drug trials). Black circles represent drug trials, grey nodes represent non-drug trials. The area of the circles is proportional to the number of trials. Connections represent comparisons in trials and the width represents the number of trials in which the comparison was present. The numbers for both circles and connections are the number of trials.
(TIF)
