# Supplementary material for: The Effects of Industry Sponsorship on Comparator Selection in Trial Registrations for Neuropsychiatric Conditions in Children
Source: PLoS One. 2013 Dec 23;8(12):e84951. doi: 10.1371/journal.pone.0084951 (PMC3871546; doi:10.1371/journal.pone.0084951)
Supplement: Table S1 — Drugs examined in neuropsychiatric trials involving children, across six conditions, ordered by prevalence in the set of 228 trials. (DOC) [file pone.0084951.s001.doc]

**Table S1. Drugs examined in neuropsychiatric trials involving children, across six conditions, ordered by prevalence in the set of 228 trials. (continues on page 2)**

| Drug Name | | Number of trials | Condition(s) | | Industry-funded trials | Combined Funding trials | Non-industry trials |
| --- | --- | --- | --- | --- | --- | --- | --- |
| methylphenidate | | 41 | Autism spectrum disorders; ADHD | | 17 | 3 | 21 |
| atomoxetine | | 21 | Autism spectrum disorders; ADHD | | 12 | 1 | 8 |
| lisdexamfetamine | | 17 | ADHD | | 10 | 5 | 2 |
| aripiprazole | | 12 | Schizophrenia; Autism spectrum disorders | | 8 | 3 | 1 |
| guanfacine | | 12 | Autism spectrum disorders; ADHD | | 7 | 3 | 2 |
| levetiracetam | | 7 | Seizure disorders | | 4 | 1 | 2 |
| fluoxetine | | 7 | Depression; Autism spectrum disorders | | 3 | 2 | 2 |
| desvenlafaxine | | 6 | Depression | | 6 | - | - |
| clonidine | | 5 | ADHD | | 5 | - | - |
| melatonin | | 5 | Seizure disorders; Autism spectrum disorders; ADHD | | - | - | 5 |
| dexmethylphenidate | | 4 | ADHD | | 3 | 1 | - |
| oxcarbazepine | | 4 | Seizure disorders; Autism spectrum disorders | | 3 | - | 1 |
| paliperidone | | 4 | Schizophrenia; Autism spectrum disorders | | 3 | 1 | - |
| zonisamide | | 4 | Migraine and other headaches; Seizure disorders | | 3 | - | 1 |
| acetylcysteine | | 3 | Autism spectrum disorders | | - | - | 3 |
| arbaclofen | | 3 | Autism spectrum disorders | | 3 | - | - |
| diazepam | | 3 | Seizure disorders | | - | 1 | 2 |
| duloxetine | | 3 | Depression | | 3 | - | - |
| molindone | | 3 | ADHD | | 3 | - | - |
| oxytocin | | 3 | Autism spectrum disorders | | - | - | 3 |
| pregabalin | | 3 | Seizure disorders | | 3 | - | - |
| risperidone | | 3 | Autism spectrum disorders; ADHD | | 1 | - | 2 |
| rizatriptan | | 3 | Migraine and other headaches | | 3 | - | - |
| sapropterin | | 3 | Autism spectrum disorders | | - | 3 | - |
| “Adderall” | | 2 | ADHD | | 1 | 1 | - |
| amitriptyline | | 2 | Migraine and other headaches | | - | - | 2 |
| asenapine | | 2 | Schizophrenia | | 2 | - | - |
| buspirone | | 2 | Autism spectrum disorders | | - | - | 2 |
| CM-AT | | 2 | Autism spectrum disorders | | 2 | - | - |
| donepezil | | 2 | Autism spectrum disorders | | - | - | 2 |
| eslicarbazepine | | 2 | Seizure disorders | | 2 | - | - |
| eszopiclone | | 2 | ADHD | | 2 | - | - |
| lacosamide | | 2 | Seizure disorders | | 1 | - | 1 |
| lorazepam | | 2 | Seizure disorders | | - | - | 2 |
| edivoxetine | | 2 | ADHD | | 2 | - | - |
| memantine | | 2 | Autism spectrum disorders | | 1 | - | 1 |
| metoclopramide | 2 | | Migraine and other headaches | - | | - | 2 |
| pozanicline | 2 | | ADHD | 2 | | - | - |
| sumatriptan | 2 | | Migraine and other headaches | 1 | | 1 |  |

**Table S**1 (continued from previous page)

| Drug Name | Number  of trials | Condition(s) | Industry-funded trials | Combined Funding trials | Non-industry trials |
| --- | --- | --- | --- | --- | --- |
| topiramate | 2 | Migraine and other headaches; Seizure disorders | 1 | - | 1 |
| treximet | 2 | Migraine and other headaches | 2 | - | - |
| valproic acid | 2 | Seizure disorders; ADHD; Autism spectrum disorders | 1 | - | 1 |
| amantadine | 1 | ADHD | - | - | 1 |
| brivaracetam | 1 | Seizure disorders | 1 | - | - |
| bumetanide | 1 | Autism spectrum disorders | - | - | 1 |
| buproprion | 1 | ADHD | - | - | 1 |
| carnitine | 1 | ADHD | - | - | 1 |
| citalopram | 1 | ADHD | - | - | 1 |
| corticotropin | 1 | Seizure disorders | - | - | 1 |
| creatine | 1 | Depression | - | 1 | - |
| cycloserine | 1 | Autism spectrum disorders | - | - | 1 |
| dexamfetamine | 1 | ADHD | - | - | 1 |
| escitalopram | 1 | Depression | 1 | - | - |
| fluconazole | 1 | Autism spectrum disorders | - | - | 1 |
| fluvoxamine | 1 | Depression | 1 | - | - |
| gabapentin | 1 | Seizure disorders | 1 | - | - |
| ganaxolone | 1 | Seizure disorders | 1 | - | - |
| glutathione | 1 | Autism spectrum disorders | - | 1 | - |
| hyperici herba | 1 | ADHD | - | - | 1 |
| ketoprofen | 1 | Migraine and other headaches | - | - | 1 |
| lamotrigine | 1 | Seizure disorders | 1 | - | - |
| lenalidomide | 1 | Autism spectrum disorders | - | 1 | - |
| mecamylamine | 1 | Autism spectrum disorders | - | - | 1 |
| metformin | 1 | Autism spectrum disorders; Schizophrenia | - | - | 1 |
| midazolam | 1 | Seizure disorders | - | - | 1 |
| minocycline | 1 | Autism spectrum disorders | - | - | 1 |
| mirtazapine | 1 | Autism spectrum disorders | - | - | 1 |
| modafinil | 1 | ADHD | 1 | - | - |
| nitrous oxide | 1 | Migraine and other headaches | - | - | 1 |
| olanzapine | 1 | Schizophrenia | 1 | - | - |
| paroxetine | 1 | Depression | 1 | - | - |
| perampanel | 1 | Seizure disorders | 1 | - | - |
| pioglitazone | 1 | Autism spectrum disorders | - | - | 1 |
| prednisolone | 1 | Seizure disorders | - | - | 1 |
| propranolol | 1 | Migraine and other headaches | - | - | 1 |
| retigabine | 1 | Seizure disorders | 1 | - | - |
| ropivacaine | 1 | Migraine and other headaches | - | - | 1 |
| rufinamide | 1 | Seizure disorders | 1 | - | - |
| selegiline | 1 | Depression | 1 | - | - |
| vigabatrin | 1 | Seizure disorders | 1 | - | - |
| vortioxetine | 1 | Depression | 1 | - | - |
| ziprasidone | 1 | Schizophrenia | - | 1 | - |
| zolmitriptan | 1 | Migraine and other headaches | 1 | - | - |
| zolpidem | 1 | ADHD | 1 | - | - |
