# Supplementary material for: The Effects of Industry Sponsorship on Comparator Selection in Trial Registrations for Neuropsychiatric Conditions in Children
Source: PLoS One. 2013 Dec 23;8(12):e84951. doi: 10.1371/journal.pone.0084951 (PMC3871546; doi:10.1371/journal.pone.0084951)
Supplement: Table S2 — The set of included trials for each of the six conditions. (DOC) [file pone.0084951.s002.doc]

**Table S2. The set of included trials for each of the six conditions.**

| ADHD | Autism spectrum disorder | Seizure disorders | Unipolar depression | Migraines and other headaches | Schizophrenia |
| --- | --- | --- | --- | --- | --- |
| NCT00029614  NCT00152750  NCT00264797  NCT00279409  NCT00280228  NCT00299234  NCT00301236  NCT00310986  NCT00318448  NCT00320528  NCT00343811  NCT00364702  NCT00367835  NCT00380692  NCT00381407  NCT00393042  NCT00402857  NCT00406354  NCT00409708  NCT00418184  NCT00428792  NCT00429273  NCT00434213  NCT00447278  NCT00471354  NCT00485797  NCT00499863  NCT00500071  NCT00500149  NCT00501293  NCT00518232  NCT00528697  NCT00541346  NCT00546910  NCT00552266  NCT00554385  NCT00556959  NCT00561340  NCT00564954  NCT00568685  NCT00573534  NCT00586157  NCT00600470  NCT00607919  NCT00626236  NCT00631280  NCT00632619  NCT00640419  NCT00641329  NCT00687609  NCT00699205  NCT00700323  NCT00704990  NCT00712699  NCT00716274  NCT00723190  NCT00723684  NCT00733356  NCT00734578  NCT00735371  NCT00754208  NCT00758160  NCT00760747  NCT00763971  NCT00764868  NCT00770627  NCT00772161  NCT00776009  NCT00778310  NCT00782080  NCT00784654  NCT00794040  NCT00794625  NCT00796302  NCT00799409  NCT00799487  NCT00802490  NCT00807222  NCT00819429  NCT00819611  NCT00834821  NCT00842127  NCT00844753  NCT00852059  NCT00856063  NCT00856973  NCT00857220  NCT00862108  NCT00867451  NCT00874536  NCT00886483  NCT00889915  NCT00904670  NCT00918567  NCT00919906  NCT00922636  NCT00930449  NCT00936299  NCT00937469  NCT00945971  NCT00947973  NCT00956605  NCT00965419  NCT00989950  NCT00997984  NCT01000402  NCT01012622  NCT01012778  NCT01017263  NCT01019252  NCT01022229  NCT01050582  NCT01052064  NCT01060150  NCT01065259  NCT01069523  NCT01073176  NCT01081132  NCT01081145  NCT01096953  NCT01099059  NCT01099072  NCT01106430  NCT01109849  NCT01124721  NCT01127646  NCT01133418  NCT01133847  NCT01137318  NCT01143701  NCT01146002  NCT01156051  NCT01161719  NCT01176136  NCT01177306  NCT01220440  NCT01228604  NCT01238822  NCT01239030  NCT01244490  NCT01274221  NCT01275378  NCT01320098  NCT01322646  NCT01328756  NCT01330693  NCT01330849  NCT01339286  NCT01340690  NCT01344044  NCT01350986  NCT01351246  NCT01352468  NCT01363544  NCT01364662  NCT01369459  NCT01388530  NCT01393574  NCT01413165  NCT01415440  NCT01416064  NCT01439126  NCT01462032  NCT01500694  NCT01542528  NCT01547702  NCT01552902  NCT01552915  NCT01583829 | NCT00277407  NCT00308074  NCT00325572  NCT00332241  NCT00335790  NCT00337571  NCT00365859  NCT00376194  NCT00378157  NCT00380692  NCT00391261  NCT00404846  NCT00406159  NCT00409747  NCT00445471  NCT00453180  NCT00467753  NCT00467818  NCT00468130  NCT00498173  NCT00503191  NCT00515320  NCT00518804  NCT00541346  NCT00549562  NCT00572741  NCT00576732  NCT00584480  NCT00614198  NCT00619190  NCT00627705  NCT00670891  NCT00676195  NCT00692315  NCT00695136  NCT00699205  NCT00725556  NCT00760812  NCT00773812  NCT00786799  NCT00787111  NCT00789932  NCT00818428  NCT00819364  NCT00844753  NCT00846547  NCT00848874  NCT00850070  NCT00870727  NCT00872898  NCT00873509  NCT00873834  NCT00881452  NCT00889538  NCT00912691  NCT00919906  NCT00926471  NCT00927030  NCT00935701  NCT00936182  NCT00943579  NCT00947700  NCT00953095  NCT00954083  NCT00954213  NCT00965068  NCT00996931  NCT00999778  NCT01008800  NCT01011764  NCT01012076  NCT01018407  NCT01031823  NCT01039792  NCT01050582  NCT01064973  NCT01078714  NCT01086475  NCT01098383  NCT01116388  NCT01141595  NCT01154777  NCT01154894  NCT01170325  NCT01177969  NCT01178385  NCT01179841  NCT01187784  NCT01190917  NCT01205282  NCT01227668  NCT01230359  NCT01233414  NCT01238575  NCT01248130  NCT01248728  NCT01250938  NCT01256060  NCT01260961  NCT01288716  NCT01302964  NCT01308749  NCT01313325  NCT01322022  NCT01337232  NCT01343511  NCT01366859  NCT01372449  NCT01379443  NCT01388179  NCT01395953  NCT01400269  NCT01417026  NCT01425918  NCT01493609  NCT01501058  NCT01502488  NCT01535508  NCT01558180  NCT01563003  NCT01565629 | NCT00326612  NCT00343096  NCT00361010  NCT00385411  NCT00393614  NCT00437281  NCT00441896  NCT00442104  NCT00448916  NCT00471744  NCT00505934  NCT00535392  NCT00552526  NCT00566254  NCT00603473  NCT00621478  NCT00639730  NCT00681239  NCT00720863  NCT00735527  NCT00741559  NCT00832884  NCT00836836  NCT00918047  NCT00918424  NCT00938912  NCT00965575  NCT00975715  NCT00988156  NCT01006811  NCT01051193  NCT01063764  NCT01079156  NCT01127165  NCT01136954  NCT01146951  NCT01161108  NCT01161524  NCT01278966  NCT01284530  NCT01292837  NCT01363154  NCT01364597  NCT01367964  NCT01378611  NCT01389596  NCT01405053  NCT01413711  NCT01431976  NCT01494584  NCT01527513  NCT01549288  NCT01575639 | NCT00283660  NCT00329199  NCT00353028  NCT00436150  NCT00461539  NCT00511810  NCT00523081  NCT00529789  NCT00531947  NCT00557427  NCT00587639  NCT00596869  NCT00611052  NCT00612313  NCT00619411  NCT00619619  NCT00628888  NCT00641368  NCT00658476  NCT00669110  NCT00680966  NCT00700609  NCT00738634  NCT00749177  NCT00776685  NCT00812812  NCT00847457  NCT00849693  NCT00849901  NCT00851006  NCT00867919  NCT00883519  NCT00891631  NCT00904891  NCT00946413  NCT00949689  NCT00951821  NCT00962598  NCT00964054  NCT01128764  NCT01137149  NCT01140464  NCT01147614  NCT01147913  NCT01159041  NCT01170520  NCT01195740  NCT01198795  NCT01201382  NCT01204346  NCT01228890  NCT01267773  NCT01341925  NCT01371708  NCT01371721  NCT01371734  NCT01372150  NCT01425905  NCT01443715  NCT01447602  NCT01491035  NCT01494831  NCT01502033  NCT01534377  NCT01537419  NCT01582581 | NCT00355394  NCT00389038  NCT00450060  NCT00488514  NCT00557544  NCT00604812  NCT00680823  NCT00777218  NCT00843024  NCT00853138  NCT00897780  NCT00963937  NCT01001234  NCT01004263  NCT01010711  NCT01016678  NCT01073787  NCT01155557  NCT01182051  NCT01211145  NCT01287052  NCT01316471  NCT01496378  NCT01581281 | NCT00257192  NCT00265382  NCT00391261  NCT00421954  NCT00465920  NCT00488319  NCT00518323  NCT00585390  NCT00757497  NCT00982020  NCT01001702  NCT01004354  NCT01009047  NCT01050582  NCT01122927  NCT01149655  NCT01190254  NCT01190267 |
