# Supplementary figures and images for: The Effects of Industry Sponsorship on Comparator Selection in Trial Registrations for Neuropsychiatric Conditions in Children
Source: PLoS One. 2013 Dec 23;8(12):e84951. doi: 10.1371/journal.pone.0084951 (PMC3871546; doi:10.1371/journal.pone.0084951)

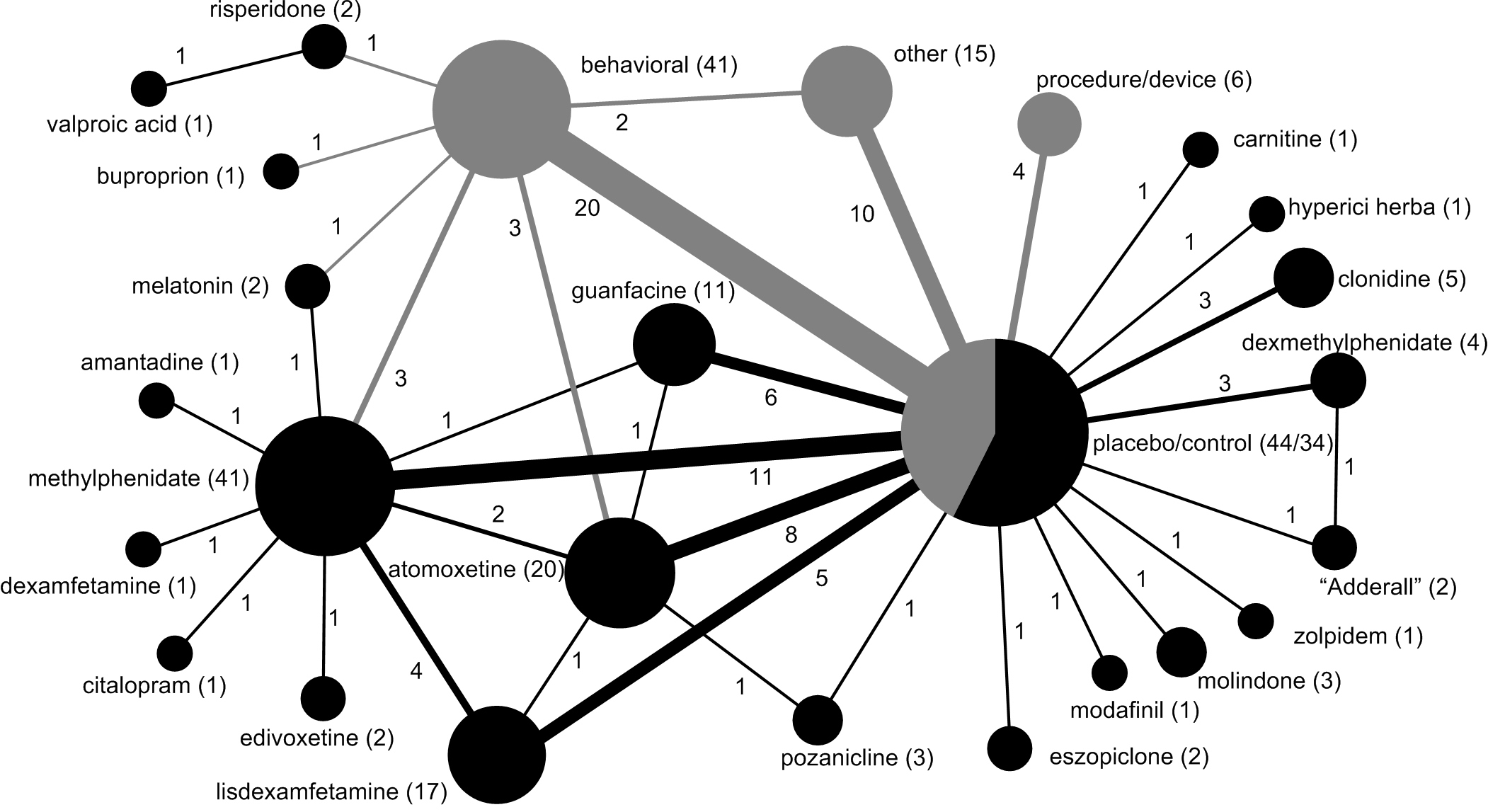

Supplement: Figure S1 — The treatment network for ADHD including interventions other than drugs. There were 162 trials that met the inclusion criteria for ADHD, illustrated here as a treatment network (inclusive of non-drug trials). Black circles represent drug trials, grey nodes represent non-drug trials. The area of the circles is proportional to the number of trials. Connections represent comparisons in trials. Connection width is proportional to the number of trials in which the comparison was present. The numbers for both circles and connections indicate trial counts. (TIF) [file pone.0084951.s003.tif]

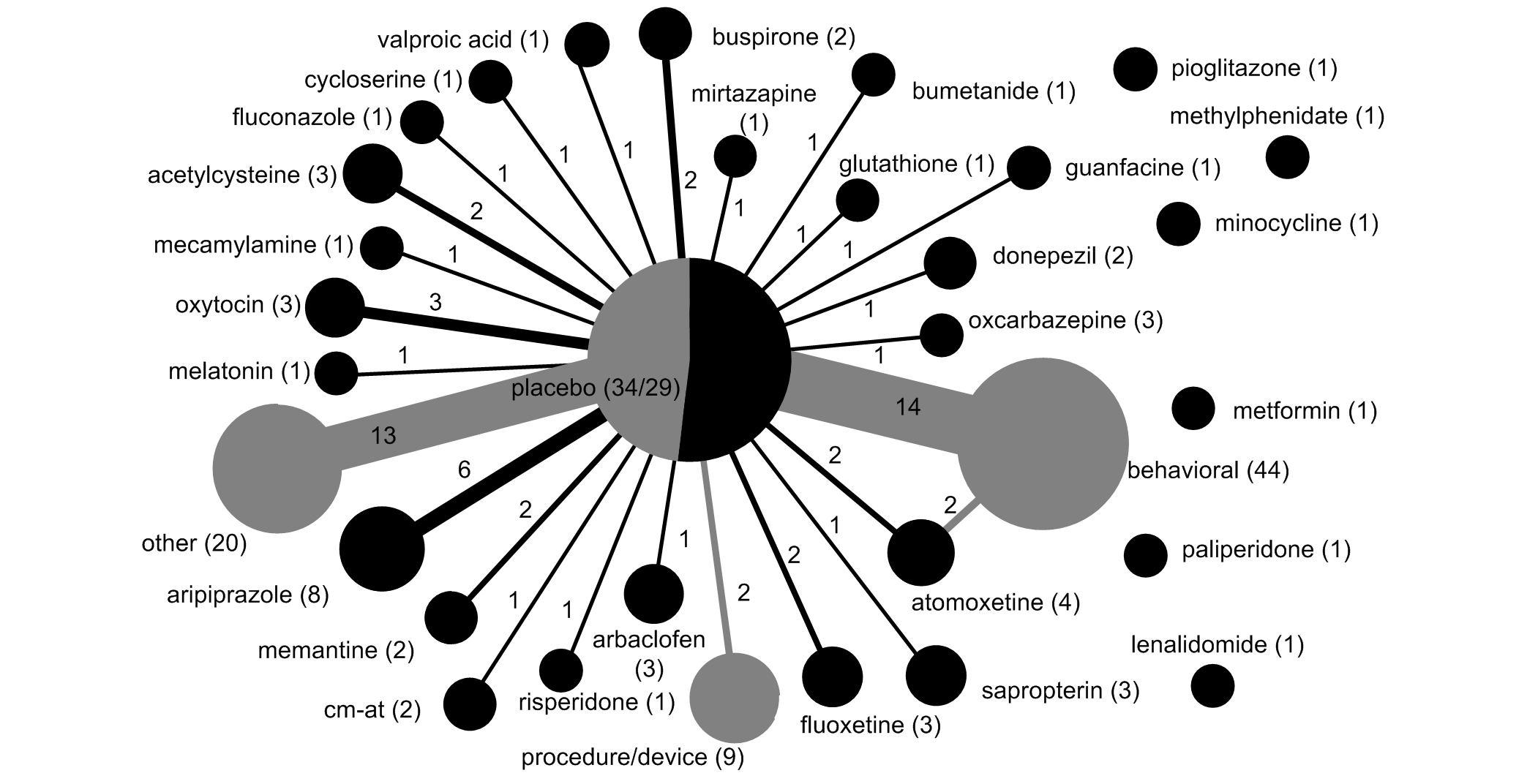

Supplement: Figure S2 — The treatment network for autism spectrum disorders including interventions other than drugs There were 116 trials that met the inclusion criteria for autism spectrum disorders, illustrated here as a treatment network (inclusive of non-drug trials). Black circles represent drug trials, grey nodes represent non-drug trials. The area of the circles is proportional to the number of trials. Connections represent comparisons in trials. Connection width is proportional to the number of trials in which the comparison was present. The numbers for both circles and connections indicate trial counts. (TIF) [file pone.0084951.s004.tif]

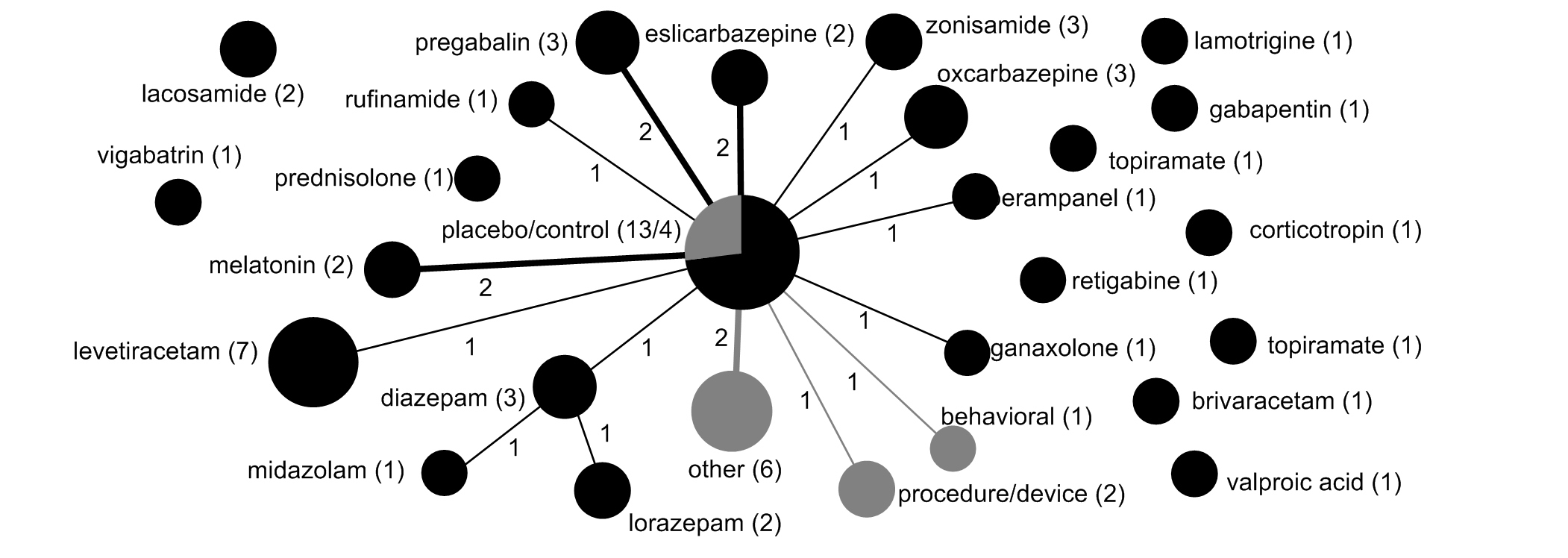

Supplement: Figure S3 — The treatment for seizure disorders including interventions other than drugs There were 49 trials that met the inclusion criteria for seizure disorders, illustrated here as a treatment network (inclusive of non-drug trials). Black circles represent drug trials, grey nodes represent non-drug trials. The area of the circles is proportional to the number of trials. Connections represent comparisons in trials. Connection width is proportional to the number of trials in which the comparison was present. The numbers for both circles and connections indicate trial counts. (TIF) [file pone.0084951.s005.tif]

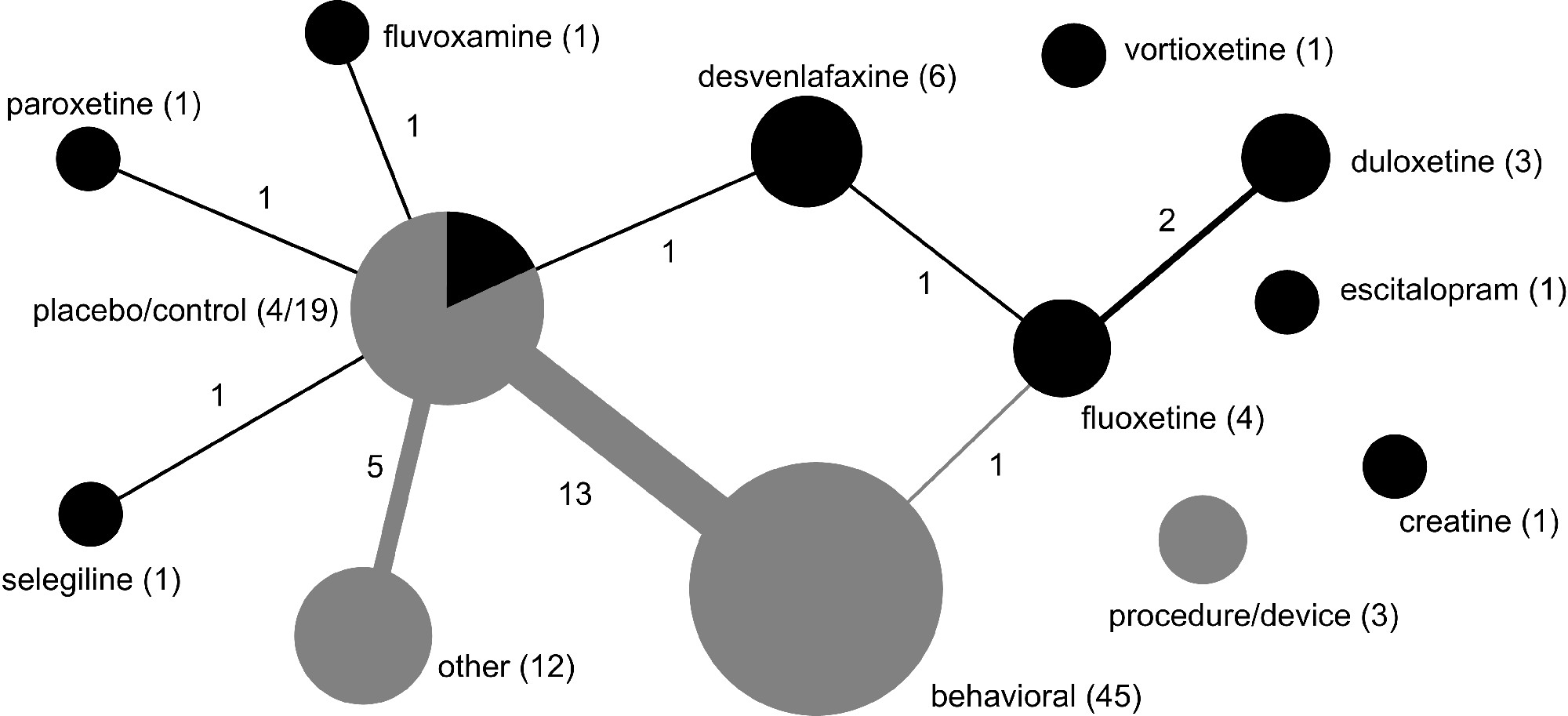

Supplement: Figure S4 — The treatment network for unipolar depression including interventions other than drugs. There were 66 trials that met the inclusion criteria for unipolar depression, illustrated here as a treatment network (inclusive of non-drug trials). Black circles represent drug trials, grey nodes represent non-drug trials. The area of the circles is proportional to the number of trials. Connections represent comparisons in trials. Connection width is proportional to the number of trials in which the comparison was present. The numbers for both circles and connections indicate trial counts. (TIF) [file pone.0084951.s006.tif]

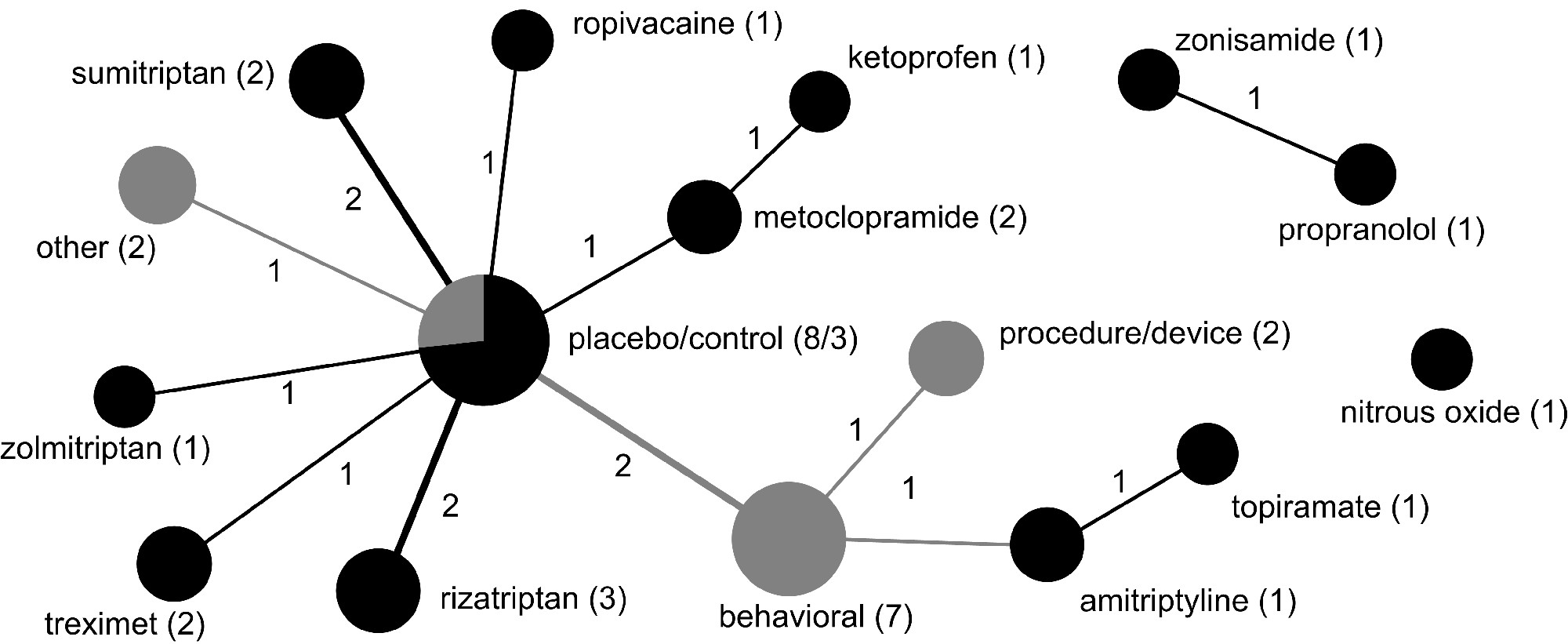

Supplement: Figure S5 — The treatment network for migraines and other headaches including interventions other than drugs. There were 24 trials that met the inclusion criteria for migraines and other headaches, illustrated here as a treatment network (inclusive of non-drug trials). Black circles represent drug trials, grey nodes represent non-drug trials. The area of the circles is proportional to the number of trials. Connections represent comparisons in trials. Connection width is proportional to the number of trials in which the comparison was present. The numbers for both circles and connections indicate trial counts. (TIF) [file pone.0084951.s007.tif]

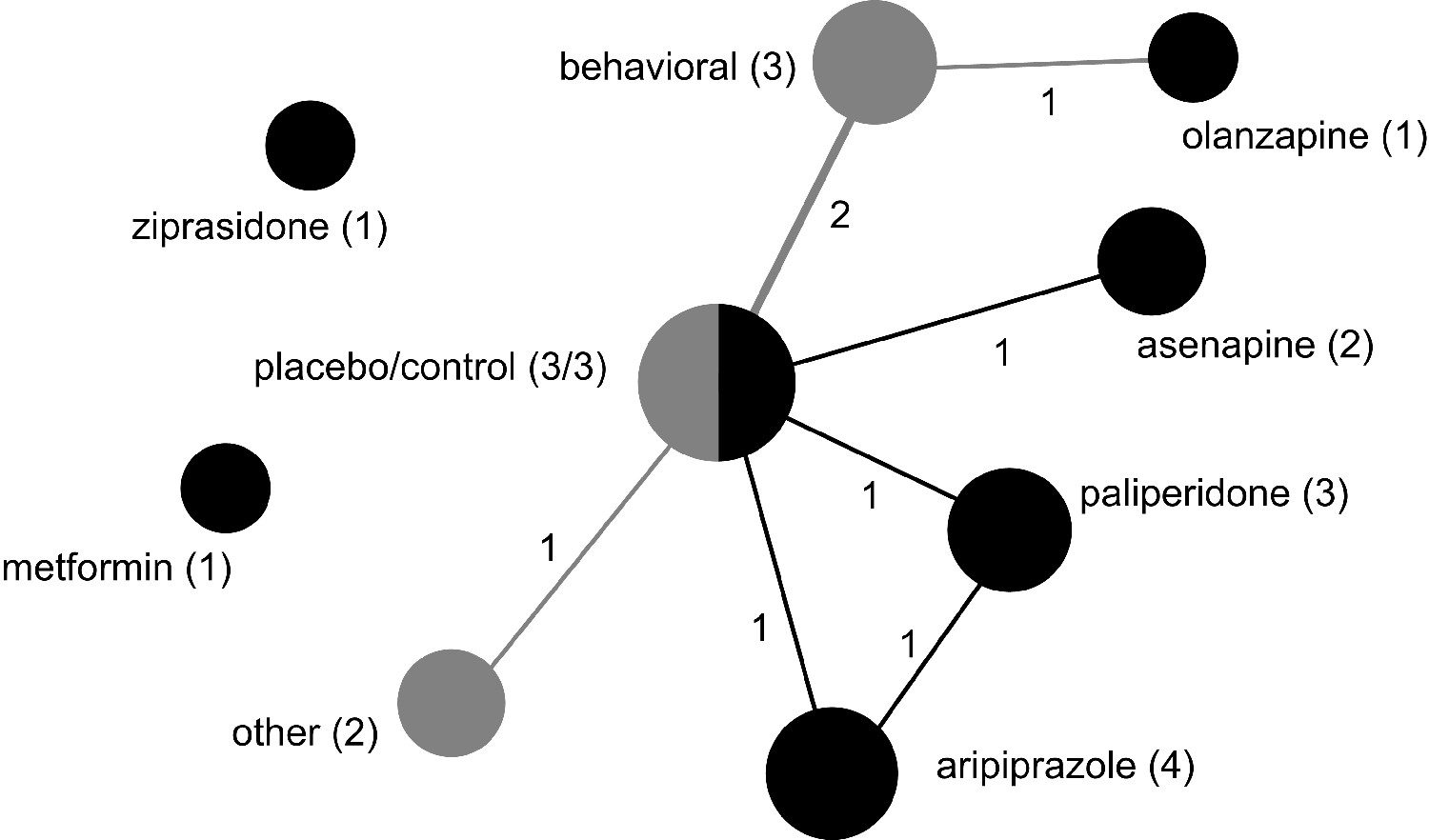

Supplement: Figure S6 — The treatment network for schizophrenia including interventions other than drugs. There were 24 trials that met the inclusion criteria for schizophrenia, illustrated here as a treatment network (inclusive of non-drug trials). Black circles represent drug trials, grey nodes represent non-drug trials. The area of the circles is proportional to the number of trials. Connections represent comparisons in trials and the width represents the number of trials in which the comparison was present. The numbers for both circles and connections are the number of trials. (TIF) [file pone.0084951.s008.tif]
